# Race-Free Equations and Cardiovascular Risk Assessment: Much Ado About Something

**DOI:** 10.5334/gh.1591

**Published:** 2026-10-23

**Authors:** George A. Mensah

**Affiliations:** 1 Center for Translation Research and Implementation Science, National Heart, Lung, and Blood Institute, National Institutes of Health, One Rockledge Centre, 6705 Rockledge Drive, Suite 402-K, Bethesda, Maryland 20892, USA

**Keywords:** Cardiovascular disease, Chronic kidney disease, race, ethnicity, risk assessment, race-based equations, estimated glomerular filtration rate, atherosclerotic cardiovascular disease risk

## Abstract

Estimated glomerular filtration rate (eGFR) is central to kidney-function assessment and cardiovascular risk prediction. The equations long used to compute it incorporated a race coefficient that artificially raised values in people of African descent. Because race is a sociocultural construct rather than a biological variable, a 2021 consensus statement recommended adoption of race-free eGFR equations—paralleling cardiology’s move from the race-based 2013 Pooled Cohort Equations to the race-free PREVENT equations. Whether removing race meaningfully alters cardiovascular risk stratification has remained largely untested in diverse populations outside North America. The HELIUS study addresses this gap, examining 21,617 Amsterdam adults from six ethnic groups, collapsed into non-African descent, African Surinamese, and Ghanaian categories. Replacing the 2009 race-corrected equation with the 2021 race-free equation lowered eGFR by roughly 9–10 mL/min/1.73 m² in the African Surinamese and Ghanaian groups, while estimates changed little in the non-African descent group. Agreement for composite chronic kidney disease (CKD) classification was near-perfect (Cohen’s κ 0.94–0.99), though driven largely by albuminuria, which is unaffected by the eGFR equation; isolating the eGFR component showed the prevalence of eGFR <60 mL/min/1.73 m² roughly doubling in Ghanaian and rising about three-quarters in African Surinamese participants, while falling in those of non-African descent. Within the SCORE2 CKD add-on model, equation choice did not materially change median risk or categorization at the population level. These reassurances are bounded by the cohort’s youth (mean age 44 years) and low burden of impaired function (1.2% with eGFR <60). As a cross-sectional study without events, HELIUS demonstrates classificational concordance but cannot establish equal predictive performance, and may not generalize to older or higher-risk populations. HELIUS nonetheless indicates that race-free estimation preserves risk categories without altering management in this low-risk cohort, while underscoring the need for rigorous, longitudinal, event-based validation across diverse global populations.

Estimated glomerular filtration rate (eGFR) is well established as a cardiovascular risk marker and an important component of contemporary guidelines for the prevention and control of cardiovascular disease (CVD) (1). The original equation for eGFR incorporated a race coefficient to mathematically account for elevated baseline creatinine concentrations linked to unverified assumptions about muscle mass (2,3). This Black race multiplier artificially enhanced eGFR values in populations of African descent. Because race is not a biological variable, there followed a decades-long debate on the appropriateness of race-based equations culminating in a 2021 consensus recommendation for the immediate adoption of race-free equations for eGFR (4). Does removing race from kidney-function estimation alter cardiovascular risk stratification, or does it change only how we justify the number? Was all the sound and fury of the debate warranted or much ado about nothing?

Race correction in clinical medicine, especially in the United States, is not a new phenomenon. In the 1850s, Samuel Cartwright used the newly invented spirometer to argue that enslaved Black people had inherently lower lung capacity—a claim grounded in slavery-era race science and later given the imprimatur of empirical support by Benjamin Gould’s 1869 anthropometric survey of Union Army soldiers (5). That episode established a durable template: a demographic ‘correction’ embedded within a measurement and presented as biology. In nephrology, it took the form of a race coefficient introduced into the 1999 Modification of Diet in Renal Disease equation—derived from the observation that Black patients had, on average, higher serum creatinine at any given measured GFR—and carried forward into the 2009 CKD-EPI equation (2,3). The rationale had migrated from overt racial ideology to defensible-appearing regression modeling, but the underlying exercise of adjusting a clinical output by race remained unchanged.

Cardiology has its own version of this history. The 2013 ACC/AHA Pooled Cohort Equations—for years the standard tool for estimating 10-year atherosclerotic cardiovascular disease risk and for guiding statin initiation—used separate race-specific equations that could materially change a patient’s estimated risk and treatment based on whether they were classified as ‘African American’ or ‘White or other’ (6). Race functioned in this setting as a coefficient, one derived from cohorts of limited diversity and without a clear mechanistic basis. That this same tool is now being replaced by the race-free PREVENT equations for cardiovascular risk—just as the race-corrected CKD-EPI equation is giving way to its race-free successor for kidney-function estimation—shows that the reappraisal HELIUS examines in chronic kidney disease (CKD) detection is already well advanced within cardiology itself (1).

Because eGFR is integral to CVD risk prediction, any change in how eGFR is computed impacts practice guidelines that incorporate it for CVD management. The current guidance that race not be used in eGFR computation therefore reaches well beyond nephrology (4). The conceptual driver of this admonition is now widely accepted: race is a socio-cultural construct and not a biological variable (7). However, the empirical question of what its removal does to risk classification has remained substantially unanswered in diverse, multiethnic populations outside the United States—which is precisely the gap HELIUS addresses (8).

Huisman et al. (8) examined 21,617 adult participants of the Healthy Life in an Urban Setting (HELIUS) study in Amsterdam drawn from six ethnic groups—Dutch, South-Asian Surinamese, African Surinamese, Ghanaian, Turkish, and Moroccan—a composition that reflects the actual diversity of a contemporary European city rather than the original Black-White dichotomy on which the original equations were built. They collapsed these six groups into three categories of distinct sociocultural and ancestral background: non-African descent, African Surinamese, and Ghanaian. The major finding shows that replacing the 2009 race-corrected equation with the 2021 race-free equation lowered eGFR by roughly 9 to 10 mL/min/1.73 m² in the African Surinamese and Ghanaian groups, while estimates in the non-African ancestry group experienced little change (Figure 1).

**Figure 1:**
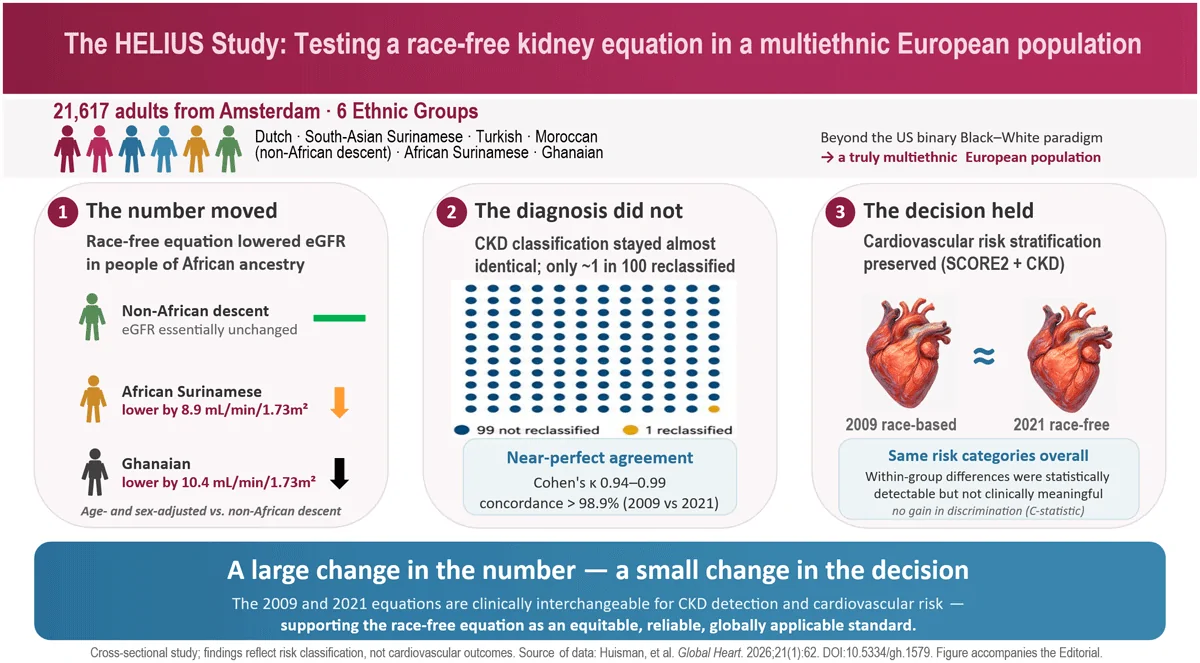
The HELIUS Study: Testing a race-free kidney equation in a multiethnic European population. Graphical summary of the effect of replacing the 2009 race-based CKD-EPI equation with the 2021 race-free equation on estimated glomerular filtration rate, chronic kidney disease classification, and cardiovascular risk stratification in the multiethnic HELIUS cohort. Figure designed by the author using OpenAI ChatGPT (GPT-5.6 Sol) for graphical layout and image generation. **Abbreviations:** CKD: chronic kidney disease; eGFR: estimated glomerular filtration rate; SCORE2: Systematic Coronary Risk Evaluation 2; US: United States.

Importantly, agreement between the two equations for CKD classification was near-perfect (Cohen’s κ 0.94–0.99) (8). But this rests on a composite definition—eGFR <60 mL/min/1.73 m² and/or albuminuria—in which albuminuria, unaffected by the eGFR equation, drives most cases. Isolating the eGFR component tells a different story; the prevalence of eGFR <60 roughly doubled in Ghanaian (1.4% to 2.9%) and rose by about three-quarters in African Surinamese participants (1.3% to 2.3%), while falling in those of non-African descent (1.2% to 0.8%) (8). Near-perfect κ reflects a young cohort with few participants near the threshold, not proof that the equations are clinically interchangeable.

Incorporation of the race-free equation into the SCORE2 CKD add-on model did not materially change median risk estimates or their categorization at the population level compared with the race-corrected equation (8). Integrating CKD measures modestly raised estimated risk overall, yet the choice of equation made little difference to stratification. That reassurance is bounded by its setting. With a mean age of 44 years and only 1.2% of participants below an eGFR of 60, this is precisely where a 9 to 10 mL/min/1.73 m² shift is least likely to move anyone across a decision boundary—inconsequential at an eGFR of 85, but decisive near the threshold of 60. Whether the same holds in older or higher-risk populations cannot be inferred from these data.

The study by Huisman et al. (8) is important because of its large sample size, genuine multi-ethnic population, standardized measurement, and analysis conducted within the contemporary European Society of Cardiology Systematic Coronary Risk Evaluation 2 (SCORE2) framework. It is one of the most informative examinations of the race-free transition outside North America, and the most relevant to cardiovascular practice in diverse settings. However, it is not without limitations. As the authors acknowledged, the cohort is young and healthy, limiting generalizability to older, high-risk, or transplant populations. Since few participants approach the eGFR threshold, it is structurally predisposed toward concordance. Single measurements of creatinine and albuminuria may also have introduced misclassification. Most importantly, classificational concordance is not the same as equal predictive performance across groups. Following no events, this cross-sectional study cannot show that the two equations predict outcomes equally well—a caution reinforced by evidence that the race-free creatinine-only equation attenuates Black versus non-Black differences in the risks of kidney failure and mortality at a given eGFR, differences that equations incorporating cystatin C preserve (9,10).

None of these limitations undermine the study’s central finding, but together they mean that HELIUS is best appreciated as strong evidence for classificational concordance and an invitation to appropriately designed, outcome-based longitudinal studies. Furthermore, HELIUS demonstrates that the race coefficient, derived and validated in US cohorts, contributed little to composite risk classification in this cohort. Removing race from the equation restores conceptual consistency to tools that cardiology and nephrology increasingly share and aligns the risk calculation with what it purports to measure using objective biological variables. As Huisman et al. (8) correctly point out, removing race from eGFR estimation may promote equitable assessment of kidney function across populations without materially affecting CKD detection or cardiovascular risk stratification.

What does the future promise in all of this? The real excitement is that the two specialties of nephrology and cardiology that once embedded race in their clinical algorithms are arriving together at risk prediction grounded in measured biology rather than social classification. HELIUS offers early evidence that this shared destination is reached without cost to the accuracy on which clinicians depend. The strong support for race-free equations in both specialties—for detecting CKD and assessing CVD risk—does not mean that race and ethnicity are no longer important in clinical medicine. Rather, it calls for the appropriate use of race and ethnicity as social and cultural constructs rather than biological variables.

For the readership of *Global Heart* and all clinician scientists interested in the prevention and control of CVD, the HELIUS study findings provide the reassurance that the transition to the race-free equation for eGFR preserves risk categories at the population level in this young, low-risk cohort, and that patient management is unlikely to be altered based on the transition alone. What the field still owes its patients and practicing clinicians is direct confirmation—rigorous, longitudinal, event-based validation of race-free cardiovascular risk prediction across the diverse global populations the World Heart Federation represents. That, and not the recalculation of a coefficient for mathematical adjustment, is the “something” worth addressing.

## Disclaimer

The views presented in this editorial are those of the author and do not necessarily reflect the views of the National Institutes of Health or the U.S. Department of Health and Human Services.
